# Glutathione impacts Hfq condensation in nitrogen-starved *Escherichia coli*

**DOI:** 10.1128/jb.00012-26

**Published:** 2026-03-23

**Authors:** Harriet R. Ellis, Volker Behrends, Gerald Larrouy-Maumus, Josh McQuail, Sivaramesh Wigneshweraraj

**Affiliations:** 1 Department of Infectious Disease, Section of Molecular Microbiology and Centre for Bacterial Resistance Biology, Imperial College London 170895, London, United Kingdom; 2 School of Medicine and Biosciences, University of West London 7364, London, United Kingdom; 3 Department of Surgery and Cancer, Institute of Reproductive and Developmental Biology, Imperial College London 170714, London, United Kingdom; 4 Department of Life Sciences and Centre for Bacterial Resistance Biology, Imperial College London 98455, London, United Kingdom; University of Notre Dame, Notre Dame, Indiana, USA

**Keywords:** Hfq, glutathione, phase condensation, nitrogen starvation, stress response

## Abstract

Nitrogen (N) is essential for bacterial growth, and adaptation to N starvation involves extensive reprogramming of metabolism and gene expression. A hallmark subcellular feature in long-term N-starved *Escherichia coli* cells is the presence of biomolecular condensates of the major bacterial RNA regulator Hfq. The Hfq condensates, which accumulate gradually during N starvation, contribute to adaptation by modulating RNA metabolism and central metabolic pathways. Metabolites play central roles in stress responses, often acting as modulators of protein function to support survival and recovery. Glutathione (GSH), a universal stress protectant, has broad roles in bacterial stress adaptation, yet its function during N starvation remains unexplored. Using a GSH-deficient mutant (Δ*gshAB*), we show that GSH is required for optimal survival and recovery from prolonged N starvation. We reveal that GSH regulates the temporal dynamics of Hfq condensation and dissipation during N starvation and recovery from N starvation, respectively, via an as-yet unknown mechanism. However, the contribution of GSH to survival during and recovery from N starvation and Hfq condensation dynamics seems to be unlinked. Overall, the results point to a role for GSH in the adaptive response to N starvation, potentially extending its canonical function as a stress protectant.

**IMPORTANCE:**

Nitrogen is a vital nutrient for bacterial growth. When nitrogen becomes scarce, bacteria must quickly adapt to survive. *Escherichia coli* forms tiny structures called Hfq condensates, which help manage genetic information flow and metabolism. Small molecules called metabolites aid bacteria in coping with stress, and one such molecule, glutathione (GSH), protects cells under various stress conditions. GSH’s role during nitrogen starvation is unknown. We used an *E. coli* mutant unable to produce GSH and found that these bacteria struggle to survive and recover from nitrogen starvation. We also discovered that GSH helps control when and how Hfq condensates form and disappear. Although these two functions seem unrelated, our study highlights GSH’s versatile role in helping bacteria adapt to nitrogen stress.

## INTRODUCTION

Nitrogen (N) is used for the biosynthesis of the building blocks of all proteins (amino acids), nucleic acids (nucleotides), and metabolites and cofactors in bacteria. As such, N is an essential component for bacterial growth. Notably, bacteria in the mammalian gut exist in a N-starved state, as they only have access to an average of one N atom for every ten carbon atoms (1). This is because mammals have evolved to keep homeostasis of the gut bacterial community by starving them of N. Similarly, many freshwater, marine, and terrestrial ecosystems, where bacteria prevail, are limited for N (2, 3). Further still, many bacterial pathogens are thought to experience N limitation in host environments, such as in the urinary tract (e.g., uropathogenic *Escherichia coli*) or *Salmonella* Typhimurium (in macrophages) (4, 5). The fact that *E. coli* respond to N deficiency by assuming the “persister phenotype” capable of evading killing by antibiotics (6) suggests a role for the adaptive response to N starvation in bacterial antibiotic recalcitrance. When bacterial systems are used for bioproduction, bacterial growth is often decoupled from bioproduction to maximize yield or metabolic pathways reprogrammed to direct production of specific biomolecules. This is often achieved by modulating N availability (7). Clearly, elucidating the mechanisms by which enteric bacteria adapt to changes in N availability is fundamental to our understanding of bacterial stress adaptation, pathogenesis, and the rational design of bacteria for bioproduction.

**Fig 1:**
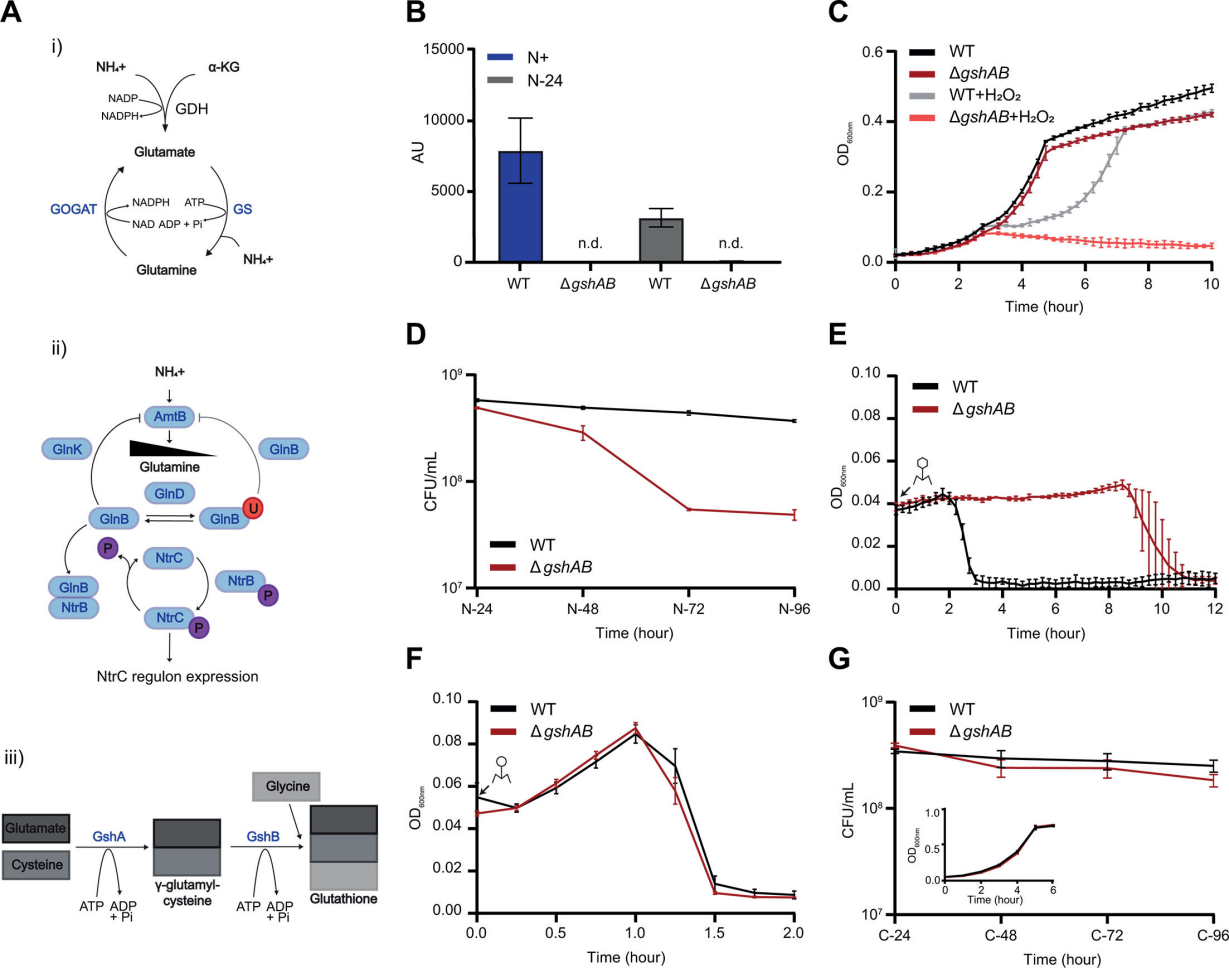
A specific role for GSH in the adaptive response to long-term N starvation. (**A**) (i) Schematic showing the assimilation of ammonium and *α*-KG into glutamate and glutamine. (ii) Schematic showing the cascade of signaling events that lead to the expression of the NtrC regulon under N starvation (the P in the purple circle indicates phosphorylation and the U in the red circle indicates uridylation). (iii) Schematic of the biosynthetic pathway that results in GSH synthesis. For panels i to iii see text for details. (**B**) Abundance of glutathione detected by targeted mass spectrometry in WT and Δ*gshAB E. coli* at *N*+ and N-24, where n.d. indicates not detected. (**C**) Growth measured by OD_600 nm_ of WT and Δ*gshAB E. coli* during N starvation, with and without the addition of H_2_O_2_ at *N*+. (**D**) Viability of WT and Δ*gshAB E.* coli during long-term N starvation measured by enumerating colony-forming units. Cultures were sampled every 24 h following N-24. (**E**) Collapse of WT and Δ*gshAB* N-24 *E. coli* cultures following infection with T7 phage. (**F**) As in (**E**), but for cultures of *N*+ *E. coli*. (**G**) Viability of WT and *ΔgshAB E. coli* during long-term C starvation was measured by enumerating colony-forming units. The inset shows the growth of WT and Δ*gshAB E. coli* under C-limiting conditions. In (**B–G**), error bars represent standard deviation (*n* = 3).

Studies from many groups, including ours, have used *E. coli* as a model system to provide a detailed picture of the gene expression changes that underpin the adaptive response to N starvation. Briefly, in *E. coli* and related bacteria, N is required for the synthesis of glutamate (for protein synthesis) and glutamine (for synthesis of nucleic acids). The enzyme glutamate dehydrogenase catalyzes the reductive amination of α-ketoglutarate (α-KG; a key intermediate of the Krebs cycle) to glutamate (Fig. 1Ai). Subsequently, glutamine synthetase catalyzes the amidation of glutamate to glutamine (Fig. 1Ai). Both glutamate dehydrogenase and glutamine synthetase use ammonium as the N source for the synthesis of glutamate and glutamine. The intracellular concentration of glutamine is the main signal for N availability in *E. coli,* and its levels are detected by the uridylyltransferase/uridylyl-removing enzyme, GlnD (for reviews see references 8–10). As shown in Fig. 1Aii, under N-replete conditions, when glutamine concentrations are high, GlnD deuridylylates GlnB. The deuridylylated form of GlnB binds to NtrB to activate its phosphatase activity and consequently dephosphorylates NtrC, thereby inactivating it. The deuridylated form of GlnK interacts with the ammonium transporter, AmtB, to inhibit ammonium uptake. Conversely, under N starvation, when the intracellular concentration of glutamine is low, GlnB and GlnK become uridylylated, which prevents the inhibition of AmtB by GlnK, thus enabling the uptake of ammonium and phosphorylation of NtrC (by NtrB), leading to expression of the NtrC regulon. The NtrC regulon is extensive and results in the transcriptional reprogramming of ~40% of all *E. coli* genes (11). Emerging results have revealed that, in addition to transcriptional reprogramming, post-transcriptional regulation of RNA, mediated by the major bacterial RNA chaperone Hfq, plays a major role in the adaptive response to N starvation in *E. coli* (12–14). Furthermore, we discovered that, during N starvation, ~50% of Hfq molecules in *E. coli* progressively assemble into a foci-like structure near the cell poles (15, 16). We term these structures “condensates” because they form through a liquid-liquid phase separation-like process and are reversible, that is, they rapidly disperse upon N replenishment (see later). Both the formation of Hfq condensates and their dispersion upon recovery from N starvation occur independently of gene expression (16), suggesting that the function of Hfq condensates extends beyond that of simply Hfq’s canonical function in facilitating the interaction between non-coding regulatory RNA molecules and their cognate mRNA targets. Indeed, in recent work, we revealed that Hfq condensates contribute to the stability of non-coding RNA and repression of sugar uptake via the phosphotransferase system during N starvation (17). Collectively, the metabolic and gene expression reprogramming that underpins the adaptive response to N starvation results in the activation of transport, catabolic, and biosynthetic systems for scavenging alternative N sources.

Glutamate, in addition to serving as a substrate for glutamine synthetase (for glutamine synthesis), also serves as a substrate for γ-glutamylcysteine synthetase (GshA), which catalyzes the first step in the two-step pathway of glutathione synthesis (Fig. 1Aiii). The second step is catalyzed by glutathione synthetase (GshB). Glutathione (GSH) is an antioxidant tripeptide (γ-glutamyl-cysteinyl-glycine) that plays essential roles in *E. coli* cellular defense and metabolism, including protecting cells against oxidative stress by scavenging reactive oxygen species, maintaining the cellular redox balance, and acting as a cofactor for various enzymes; GSH also participates in the detoxification of harmful compounds (heavy metals) and helps maintain protein thiols in their reduced state (reviewed in reference [18]). Given that GSH is an abundant nitrogenous compound and adaptation to N starvation in *E. coli* and related bacteria is primarily a scavenging response for alternative N sources, in this study, we explored GSH’s role in the adaptive response to N starvation.

## RESULTS

### GSH has a specific role in the adaptive response to long-term N starvation

To characterize the Δ*gshAB* mutant bacteria under N starvation, we grew a batch culture of *E. coli* strain MG1655 in a highly defined minimal growth medium with a limiting amount of ammonium chloride as the sole N source (19). Under these conditions, when ammonium chloride in the growth medium runs out (N-), the bacteria enter a state of N starvation and become growth attenuated. In control experiments, we used liquid chromatography-electrospray ionization mass spectrometry to confirm that GSH was indeed not present in Δ*gshAB* bacteria (Fig. 1B). We also observed that the GSH levels in wild-type (WT) bacteria decrease by ~33% following 24 h of N starvation (N-24) compared to N-replete (*N*+) condition, potentially suggesting that GSH is broken down during N starvation (see later). Hydrogen peroxide (H_2_O_2_) is a major contributor to oxidative damage and is effectively neutralized by GSH. In the absence of H_2_O_2_, the growth dynamics of Δ*gshAB* bacteria did not markedly differ from that of WT bacteria during N-replete conditions, and both strains became growth attenuated around the same time, suggesting that the dynamics of N assimilation under our conditions is unaffected in Δ*gshAB* bacteria (Fig. 1C). Furthermore, as shown in Fig. 1C, the addition of H_2_O_2_ at *N*+ compromised, but did not fully inhibit, the growth of WT bacteria. Conversely, H_2_O_2_ addition adversely affected the growth of Δ*gshAB* bacteria, affirming the importance of GSH in defense against oxidizing agents (Fig. 1C). Notably, the absence of GSH did not initially impair the survival of the bacteria, as at N-24, the proportion of viable cells in the WT and Δ*gshAB* population did not differ (Fig. 1D). However, as N starvation persisted, the proportion of viable cells in the Δ*gshAB* population began to decline, and by N-96, only ~13% were viable compared to those in the WT population (Fig. 1D). The compromised viability of *E. coli* during prolonged N starvation suggests that long-term adaptive metabolism is perturbed when the bacteria cannot synthesize GSH.

The efficacy by which bacteriophages infect and replicate in bacteria can serve as an indicator of bacterial metabolic “health.” Therefore, we measured how quickly the prototypical *E. coli* bacteriophage T7 replicated and caused the collapse of WT and Δ*gshAB* cultures from N-24. As shown in Fig. 1E, following the addition of T7, the culture of N-24 Δ*gshAB* bacteria collapsed ~6.5 h later than that of the WT bacteria. This lag in culture collapse was not seen when WT and Δ*gshAB* cultures were infected with T7 before N starvation (*N*+) (Fig. 1F). To understand whether the long-term starvation survival defect of Δ*gshAB* bacteria is specific to N starvation, we conducted experiments in growth media with excess N source, but limiting glucose (the sole carbon [C] source). Therefore, growth attenuation in this medium correlates with C starvation. As shown in Fig. 1G (*inset*), the growth dynamics of Δ*gshAB* bacteria did not markedly differ from that of WT bacteria during C-replete conditions. However, unlike under N starvation, the survival dynamics of Δ*gshAB* and WT bacteria did not differ during prolonged C starvation (Fig. 1G), suggesting that GSH has some specific role in the adaptive response to long-term N starvation in *E. coli*.

### GSH regulates the temporal dynamics of Hfq condensation during N starvation

**Fig 2:**
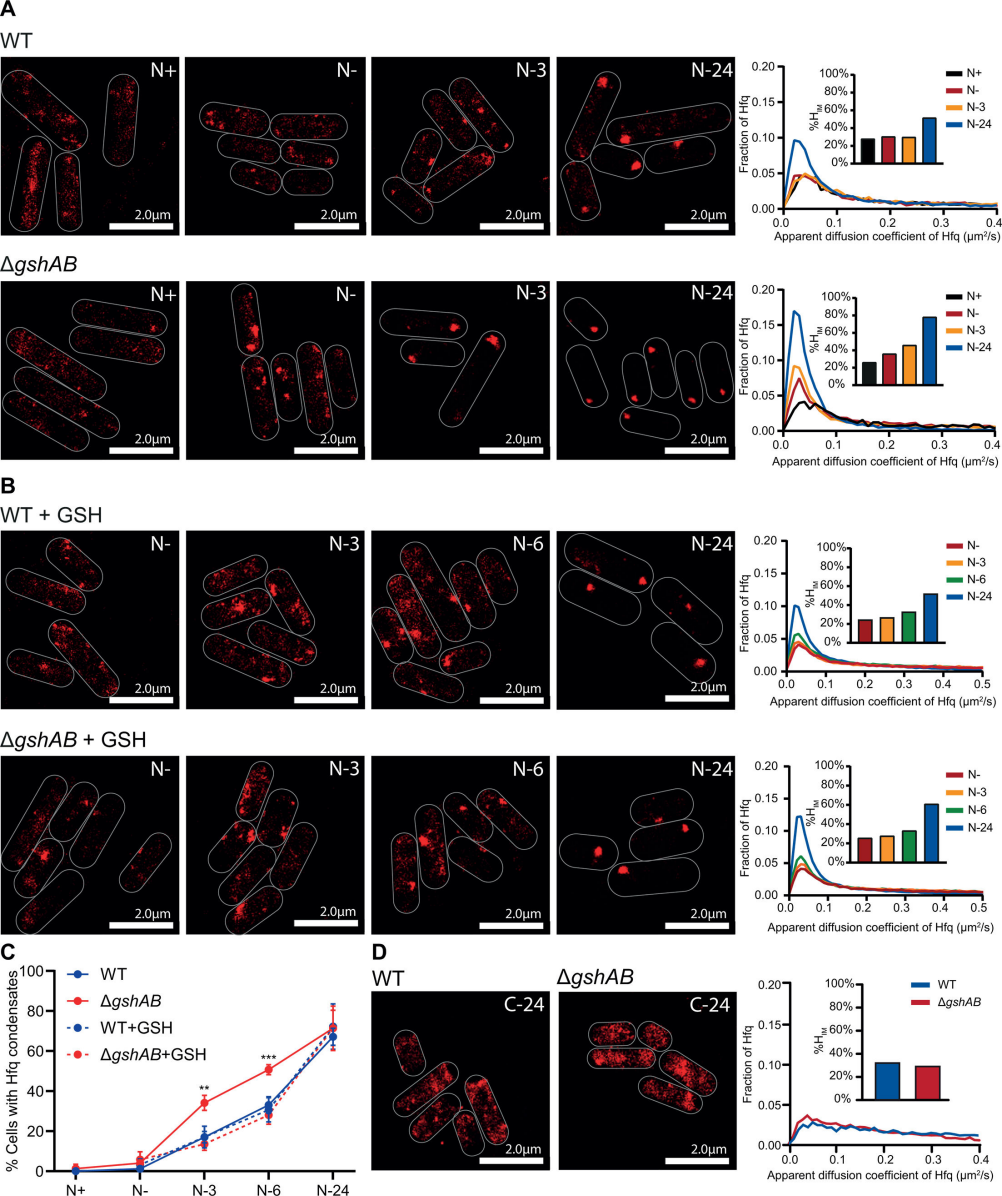
The regulation of the temporal dynamics of Hfq condensation by GSH during N starvation. (**A**) Representative PALM images of Hfq in WT (top) and Δ*gshAB* (bottom) *E. coli* as a function of time during N starvation. Images taken at indicated timepoints. Graphs show the distribution of apparent diffusion coefficients of Hfq molecules at the different sampling time points, inset bar graphs show corresponding %H_IM_ values. (**B**) As in (**A**) but with addition of 1 mM of GSH at *N*+. (**C**) Graph showing the proportion of cells with detectable Hfq condensates during N starvation as in (**A**) and (**B**). Error bars represent standard deviation (*n* > 3). Statistical analysis performed by two-way ANOVA with Dunnett’s multiple comparisons (***, *P* < 0.001; **, *P* < 0.01). (**D**) As in (**A**) but for 24 h C-starved WT and Δ*gshAB* bacteria (C-24).

We previously reported that Hfq condensation is a hallmark subcellular response to long-term N starvation: Hfq condensates are not detected at the onset of N starvation (i.e., N-) but form progressively as N starvation ensues, with early condensates appearing 3 h into N starvation (N-3) and well-defined condensates becoming clearly detectable after ~6 (N-6). Given that Δ*gshAB* bacteria display compromised survival under long-term N starvation, we used the dynamics of Hfq condensation as a proxy to assess cellular adaptation and used photoactivated localization microscopy (PALM) combined with single-molecule tracking of individual Hfq molecules in WT and Δ*gshAB* bacteria to measure Hfq condensation during N starvation. As a quantitative parameter to measure Hfq condensation, we calculated the proportion of total Hfq molecules with an apparent diffusion (D*) less than 0.08 μm^2^/s, which we had previously defined as the “immobile” population of Hfq molecules (%H_IM_) (16). The %H_IM_ was calculated based on all trajectories of Hfq in 50–200 bacterial cells within a given field of view. Alongside this measure, the proportion of cells containing detectable condensates was determined. As shown in Fig. 2A and C, we did not detect any differences in %H_IM_ or the proportion of cells with condensates between WT and Δ*gshAB* bacteria at *N*+. However, upon N run-out (N-) and as N starvation set in for a prolonged period (N- → N-24), the %H_IM_ in Δ*gshAB* bacteria was consistently higher than in WT (Fig. 2A), with the proportion of cells containing condensates by N-3 being significantly greater in Δ*gshAB* bacteria than in WT bacteria (Fig. 2C). The %H_IM_ value continued to be greater in Δ*gshAB* bacteria throughout N starvation; however, the proportion of cells with condensates was comparable to WT by N-24.

To establish that the observed differences in Hfq condensation are linked to GSH, we exogenously added 1 mM GSH to both WT and Δ*gshAB* bacteria at *N*+ and monitored Hfq condensation by PALM. We opted for chemical complementation over genetic complementation because (i) GSH is actively imported by *E. coli,* thus allowing better restoration of cellular homeostasis and (ii) plasmid-based expression of *gshAB* genes could lead to excess GSH build-up in cells, potentially altering cellular physiology. As shown in Fig. 2B and C, exogenous addition of GSH reverted the temporal dynamics of Hfq condensation in Δ*gshAB* bacteria to that seen in WT bacteria—with both %H_IM_ and the proportion of cells with condensates being indistinguishable from WT. In previous work, we showed that Hfq condensates are absent in *E. coli* that have been specifically C starved for 24 h (C-24). Therefore, to investigate whether the enhanced propensity to form condensates is a general property of Δ*gshAB* bacteria or, like in WT bacteria, a specific response to long-term N starvation, we compared the Hfq condensation dynamics in WT and Δ*gshAB* C-24 bacteria. As shown in Fig. 2D, we failed to detect distinct Hfq condensates in WT and Δ*gshAB* C-24 bacteria. We conclude that GSH impacts Hfq condensation in N-starved *E. coli*, and its absence alters the temporal dynamics of Hfq condensation, which is indicative of perturbed cellular adaptation during prolonged N starvation.

### The properties of Hfq condensates that form in WT and Δ*gshAB* bacteria are similar

**Fig 3:**
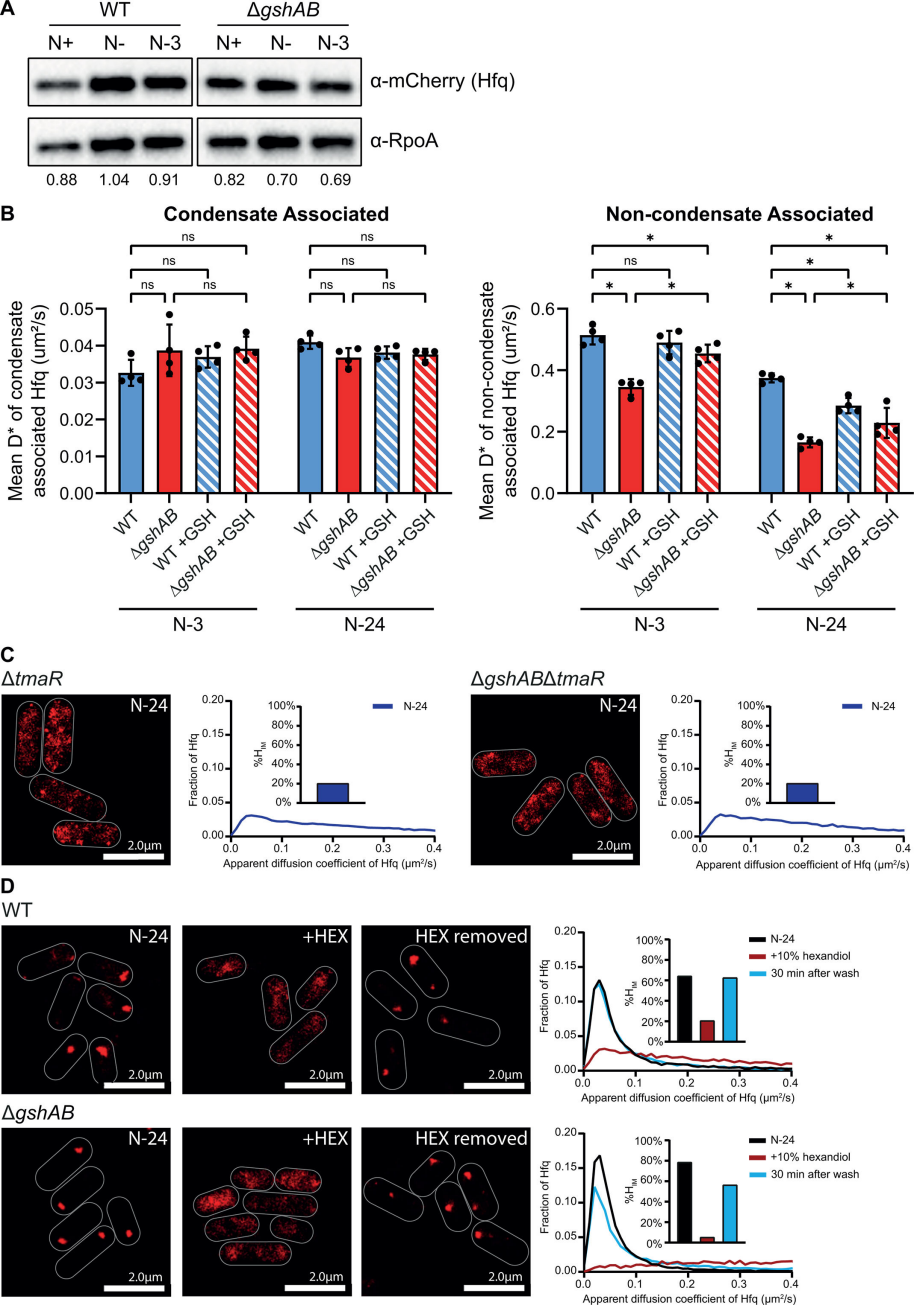
Hfq condensates in WT and Δ*gshAB* bacteria are alike. (**A**) A representative immunoblot of whole-cell extracts of WT and Δ*gshAB E. coli* with Hfq translationally fused with PAmCherry*,* sampled at *N*+, N−, and N-3, and probed with anti-mCherry antibody (for Hfq) and anti-RpoA antibody (loading control). The ratio of mCherry to RpoA signal is shown below the blot. (**B**) Graphs showing the mean apparent diffusion coefficient (D*) of molecules of Hfq found either wholly within (left) or outside (right) the Hfq condensates. Error bars represent standard deviation (*n* = 4). Statistical analysis performed by two-way ANOVA with Dunnett’s multiple comparisons (*, *P* < 0.05; ns, not significant). (**C**) Representative PALM images of Hfq in Δ*tmaR* (left) and Δ*gshABΔtmaR* (right) *E. coli* at N-24. Graphs show the distribution of apparent diffusion coefficients of Hfq molecules at the different sampling time points, inset bar graphs show corresponding %H_IM_ values. (**D**) As in (**C**), but for WT and Δ*gshAB E. coli* at N-24, following treatment with 10% hexanediol (HEX), and 30 min following the removal of HEX.

We previously reported that Hfq condensation during long-term N starvation is dependent on the PTS regulator TmaR and that Hfq condensates form by a process analogous to liquid-liquid phase separation (LLPS) (15). To study the properties of the Hfq condensates that form in Δ*gshAB* bacteria, we initially sought to exclude the possibility that the altered Hfq condensation dynamics in Δ*gshAB* bacteria was not due to any aberrant increase in Hfq protein levels. Indeed, as shown in Fig. 3A, Hfq protein levels in WT and Δ*gshAB* bacteria were very similar at *N*+, N–, and, importantly, at N-3, when the difference in Hfq condensation is detected between WT and Δ*gshAB* bacteria.

By N-3, ~15% of WT cells and ~40% of Δ*gshAB* cells contained Hfq condensates (Fig. 2C), and by N-24 ~70% of WT and Δ*gshAB* cells contained Hfq condensates. To determine whether the Hfq condensates formed in Δ*gshAB* cells displayed different properties from those in WT cells, we calculated the average apparent diffusion coefficient (D*; μm^2^/s) of Hfq molecule-trajectories found wholly *within* Hfq condensates at N-3 and N-24. As shown in Fig. 3B, we did not detect any differences in the average D* of Hfq condensate-associated Hfq molecules in WT and Δ*gshAB* bacteria at either time point, both in GSH-untreated and -treated samples (see above, Fig. 2B)—suggesting that the diffusion dynamics, and thus molecular density, of the condensates themselves are comparable between WT and Δ*gshAB* bacteria. Conversely, we further calculated the average D* of Hfq molecule trajectories found wholly *outside* of the Hfq condensates. We observed a significant decrease in average D* in Δ*gshAB* bacteria at both N-3 and N-24, as compared to WT bacteria (Fig. 3B). Notably, treatment of Δ*gshAB* bacteria with GSH (as in Fig. 2B) increased the average D* of Hfq molecules not associated with condensates and was closer to that seen in WT bacteria. By N-24, while GSH treatment still led to an increase in D* of Hfq molecules not associated with condensates (as compared to untreated Δ*gshAB*), this increase was not as substantial as at N-3; furthermore, GSH treatment of WT bacteria led to a partial reduction in average D*. Overall, the data suggest that potential differences in cytoplasmic diffusibility, that is, lower diffusibility in Δ*gshAB* bacteria, could account for the earlier formation of Hfq condensates in the absence of GSH. However, given that the condensation dynamics of other proteins known to form condensates, such as the RNA polymerase or RNase E, did not change in Δ*gshAB* bacteria (Fig. S1), we suggest that the rapid formation of Hfq condensates in Δ*gshAB* bacteria (e.g., at N-3) during N starvation is not exclusively due to reduced cytoplasmic diffusibility in the absence of GSH.

To further determine any difference in properties between Hfq condensates in Δ*gshAB* bacteria, we monitored Hfq condensation dynamics in Δ*gshAB* bacteria devoid of TmaR (Δ*gshABΔtmaR*), and the sensitivity of the Hfq condensates in Δ*gshAB* bacteria to hexanediol (HEX), an aliphatic alcohol that disrupts LLPS condensates. As in Δ*tmaR* bacteria, we did not detect any Hfq condensates in N-24 (Δ*gshABΔtmaR*) bacteria (Fig. 3B), suggesting that TmaR remains a requirement for Hfq condensation in Δ*gshAB* bacteria. Furthermore, the Hfq condensates in N-24 WT and Δ*gshAB* bacteria were both sensitive to HEX and reformed upon removal of HEX, suggesting that the altered temporal Hfq condensation dynamics in Δ*gshAB* bacteria was not due to any aberrant aggregation of Hfq (Fig. 3C). Although we are unable to explain why the presence of HEX reduced the %H_IM_ in Δ*gshAB* bacteria far below its basal levels seen in WT bacteria (Fig. 3C), our results collectively show that the properties of Hfq condensates in WT and Δ*gshAB* bacteria are nonetheless alike.

### GSH affects the temporal dynamics of Hfq condensation during N starvation without needing to be catabolized

**Fig 4:**
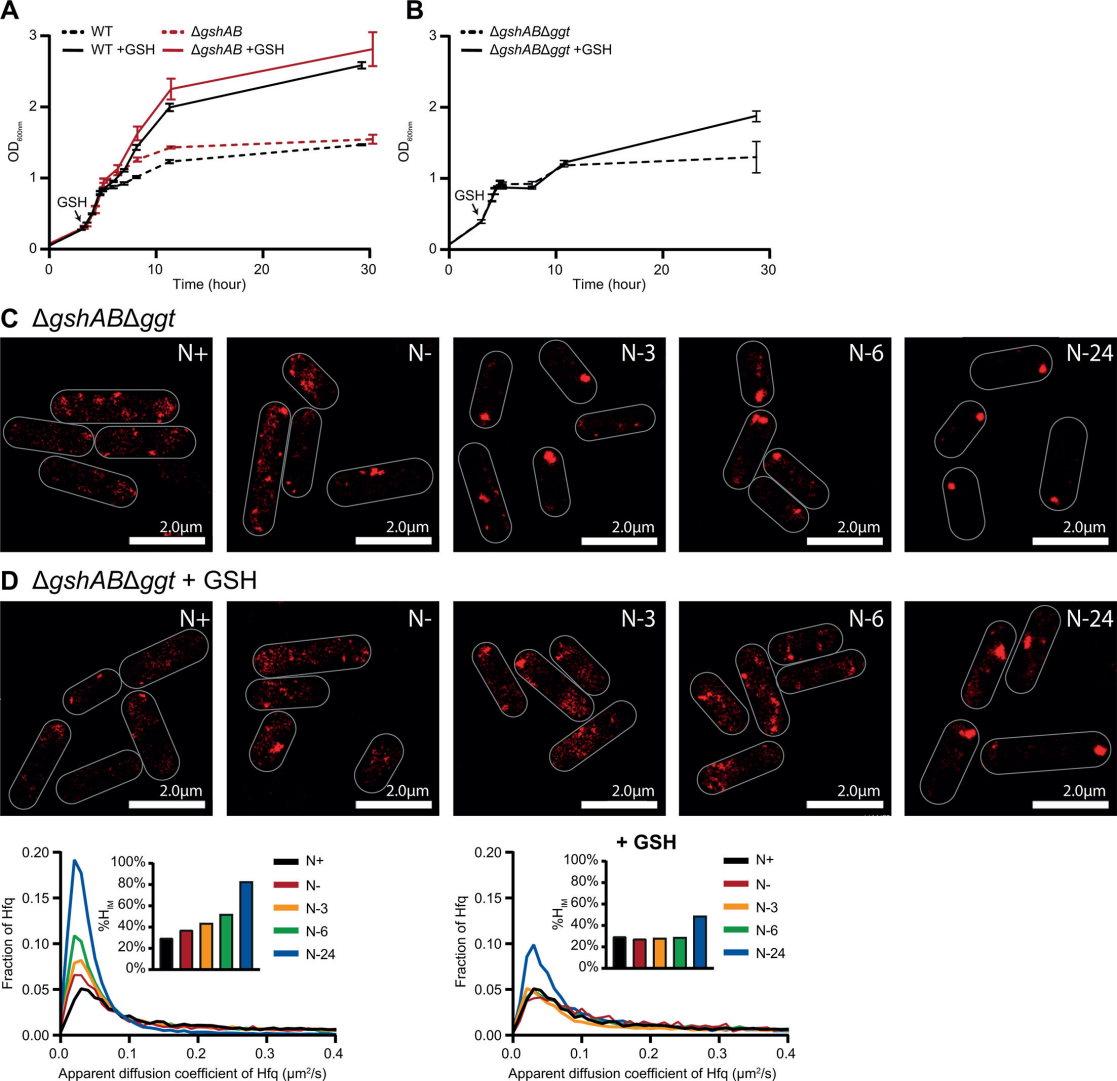
GSH does not affect Hfq condensation dynamics by serving as an N source. (**A**) Growth of WT and Δ*gshAB E. coli,* with and without the addition of 1 mM GSH at *N*+, as measured by OD_600 nm_. Error bars represent standard deviation (*n* = 3). (**B**) As in (**A**), but of Δ*gshAB* and Δ*gshAB*Δ*ggt E. coli*. (**C**) Representative PALM images of Hfq in Δ*gshAB*Δ*ggt E. coli* as a function of time during N starvation. (**D**) As in (**C**), but with the addition of 1 mM GSH at *N*+. Graphs show the distribution of apparent diffusion coefficients of Hfq molecules at the different sampling time points for (**C**) (left) and (**D**) (right), inset bar graphs show corresponding %H_IM_ values.

Under our experimental condition, bacteria enter growth attenuation at N− (~5 h after inoculation); however, we observed that when GSH was added at *N*+, growth only began to slow down ~10 h after inoculation, at a much greater OD_600 nm_ than untreated cultures (Fig. 4A). We thus considered whether GSH is catabolized as an alternative N source when the main (preferred) N source, ammonium chloride, has run out. To test this, we deleted *ggt*, which encodes γ-glutamyl transpeptidase, the enzyme primarily responsible (to the best of our knowledge) for degrading extracellular GSH, in WT and Δ*gshAB* bacteria. We then compared the growth of GSH-untreated and -treated (at *N*+) Δ*gshAB*Δ*ggt* bacteria during N starvation, with that of WT and Δ*gshAB* bacteria. The growth dynamics of GSH-untreated WT (Fig. 4A, black dotted line), Δ*gshAB* (Fig. 4A, red dotted line), and Δ*gshABΔggt* (Fig. 4B, black dotted line) were similar. Conversely, whereas exogenous addition of GSH stimulated the growth of WT (Fig. 4A, black solid line) and Δ*gshAB* bacteria (Fig. 4A, red solid line) substantially beyond that of untreated bacteria (with initial growth continuing to a final OD_600 nm_ of ~2.5), GSH treatment of Δ*gshABΔggt* bacteria (Fig. 4B, black solid line) did not substantially prolong the initial growth phase. We do note, however, that GSH treatment of Δ*gshABΔggt* bacteria did result in a gradual increase in OD_600 nm_ over the prolonged period of N-starvation, as compared to the untreated cultures, suggesting that GSH somewhat stimulated the growth of Δ*gshABΔggt* bacteria (Fig. 4B). This potentially implies that some of the exogenously added GSH is broken down in N-starved *E. coli* in a γ-glutamyl transpeptidase-independent manner and used as an N source later during starvation, supporting the growth.

Next, we considered whether treatment with GSH would still restore WT Hfq condensation dynamics to Δ*gshAB* bacteria in the absence of the ability to utilize it as an effective N source. The temporal dynamics of Hfq condensation did not differ between the Δ*gshAB* and Δ*gshABΔggt*, with Hfq still forming condensates earlier during N starvation in Δ*gshAB* and Δ*gshABΔggt* compared to in WT bacteria (compared Fig. 2A and 4C). Notably, however, the exogenous addition of GSH to Δ*gshABΔggt* bacteria still reverted the temporal dynamics of Hfq condensation to that seen in WT bacteria (compare Fig. 2A and 4D), demonstrating that the addition of GSH reverts Hfq condensation dynamics back to that of WT bacteria without having to be broken down. We therefore conclude that GSH affects the condensation dynamics of Hfq during N starvation without needing to be catabolized.

### The absence of GSH compromises the growth recovery from N starvation

**Fig 5:**
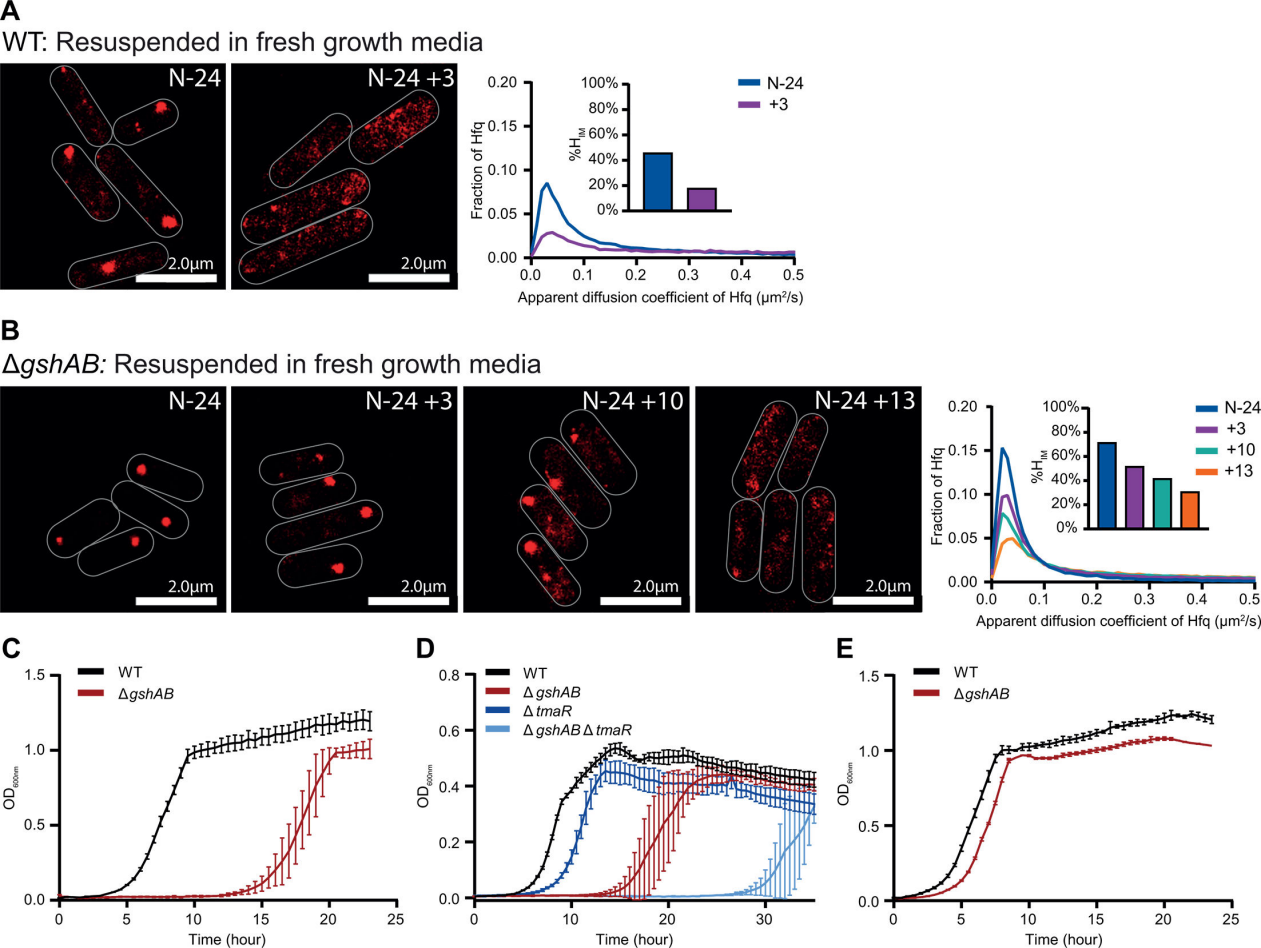
GSH-deficient bacteria experience compromised growth recovery. (**A**) Representative PALM images of Hfq in WT *E. coli* at N-24 and 3 h following resuspension into growth-permissive media (*N*+/C+). Graphs show the distribution of apparent diffusion coefficients of Hfq molecules at the different sampling time points, inset bar graphs show corresponding %H_IM_ values. (**B**) As in (**A**) but for Δ*gshAB E. coli* imaged 3, 10, and 13 h post-resuspension into growth-permissive media. (**C**) Growth recovery of WT and Δ*gshAB E. coli* from N-24 following subculturing into growth-permissive media. Error bars represent standard deviation (*n*= 3). (**D**) As in (**C**), but for WT, Δ*gshAB,* Δ*tmaR,* and Δ*gshAB*Δ*tmaR E. coli*. (**E**) As in (**C**), but for WT and Δ*gshAB E. coli* initially grown to C-24 and subsequently subcultured into growth-permissive media.

If the N-24 bacteria are resuspended into fresh media, the Hfq condensates disperse and growth resumption occurs (16). To better understand how GSH influences Hfq condensation, we monitored the temporal dispersion dynamics of Hfq condensates and growth recovery of Δ*gshAB* bacteria. As shown in Fig. 5A, and as expected, the resuspension of N-24 WT bacteria into growth-permissive media (replete with N and C) led to the dispersion of Hfq condensates, and ~3 h after resuspension the Hfq condensates were fully dispersed (also see reference [16]). When Δ*gshAB* bacteria were resuspended in growth-permissive media, even after ~10 h following resuspension, the Hfq condensates remained present, but eventually dispersed ~13 h after resuspension (Fig. 5B). We observed that the growth recovery of WT and Δ*gshAB* bacteria in growth-permissive media significantly differed and appeared to correlate with the dispersion of the Hfq condensates. As shown in Fig. 5C, the lag time to growth recovery (t_lag_) was ~3 h for WT bacteria and ~13 h for Δ*gshAB* bacteria. Thus, we considered whether the dispersion of the Hfq condensates is linked with (i.e., determines) growth recovery. Hence, we conducted experiments with Δ*gshABΔtmaR* bacteria in which Hfq condensates do not form (Fig. 3C). As shown in Fig. 5D, the t_lag_ between WT and Δ*tmaR* bacteria differed by ~2.6 h, but this difference increased to ~17.1 h in the Δ*gshAB* background (i.e., Δ*gshABΔtmaR* bacteria). Consistent with a specific role for GSH in adaptive response to long-term N starvation (Fig. 1), the t_lag_ between C-24 WT and Δ*gshAB* bacteria only differed by ~1.44 h (Fig. 5E). We conclude that the inability of *E. coli* to synthesize GSH compromises its ability to recover specifically from long-term N starvation. We further conclude that the compromised growth recovery and the altered dispersion dynamics of Hfq condensation are mutually exclusive properties of N-starved *E. coli* devoid of GSH, as the compromised recovery occurs even in the complete absence of Hfq condensate formation.

## DISCUSSION

Bacterial adaptive responses to environmental stresses are often investigated under acute exposure conditions. Consequently, the metabolic and gene expression changes that underpin adaptation during prolonged stress remain poorly understood. In *E. coli* and related bacteria, GSH is among the most abundant metabolites, primarily functioning as an antioxidant and detoxifying agent (18). *E. coli* strains lacking GSH exhibit normal growth rates even in minimal media, yet display heightened sensitivity to oxidative stress. Through characterization of an *E. coli* mutant deficient in both GSH biosynthetic enzymes (Δ*gshAB*), we have uncovered a role for GSH in the adaptive mechanisms that support survival under, and recovery from, prolonged N starvation. Notably, GSH deficiency does not impair adaptation to prolonged C starvation, highlighting a specific requirement for GSH in the N starvation adaptive response, which is likely to be distinct from its canonical functions as an antioxidant/detoxifying agent.

Biomolecular condensates have emerged as a key mechanism for subcellular organization in bacteria, enabling spatial and temporal regulation of cellular processes (20–22). Among these, condensates formed by Hfq are a hallmark feature of stress-associated biomolecular assemblies (15–17). During N starvation, Hfq condensates progressively appear as the stress intensifies. Our new findings reveal that in *E. coli* cells lacking GSH, Hfq condensates form significantly earlier, suggesting that GSH-deficient cells enter a heightened stress state more rapidly than their WT counterparts. It is possible that accelerated Hfq condensation in Δ*gshAB* bacteria during N starvation is due to increased redox stress, which, in turn, can induce polyP synthesis (23), which has been shown to serve as a scaffold for Hfq condensation (24). However, as Hfq condensation is not induced by H_2_0_2_ (25), we propose that it is unlikely that Hfq condensation is a direct response to redox stress that incurs in cells devoid of GSH, but a direct response linked to N starvation. Our results indicate that the Hfq condensates that form in WT and ∆*gshAB* bacteria are alike (Fig. 3). As Hfq condensation depends on TmaR condensation in N-starved *E. coli* (17), it is possible that GSH directly affects TmaR condensation dynamics. However, this is difficult to measure as TmaR condensates are already present in most cells even prior to the onset of N starvation when Hfq condensates are absent (17). Building on our previous findings that Hfq condensates can be induced by the exogenous addition of α-KG (17), our new results suggest that the condensation dynamics of Hfq (and potentially other proteins) are modulated by metabolite fluxes during stress adaptation. These observations underscore the possibility that specific metabolites, in addition to nucleic acids, can influence the formation and timing of biomolecular condensates in response to stresses to spatiotemporally organize and concentrate specific cellular processes in the stressed cell.

The heterotypic composition of biomolecular condensates and the pleiotropic nature of the proteins involved in their formation make it challenging to assign discrete functions to these assemblies. Hfq condensates emerge progressively during N starvation in *E. coli* and disperse upon growth recovery from N starvation (16). Therefore, it is conceivable that they are likely to play roles in both adaptation to nutrient stress and recovery. Indeed, our findings appear to show that in GSH-deficient bacteria, Hfq condensates disperse significantly more slowly than in WT cells, and that the timing of dispersal coincides with the recovery of growth following N starvation. However, this correlation does not imply causality. In fact, our data suggest that Hfq condensates do not directly contribute to growth recovery. Rather, consistent with our previous results (17), they appear to contribute to post-transcriptional and metabolic regulation during adaptation to prolonged N starvation *per se*.

Our findings suggest a specific role for GSH in facilitating growth recovery of N-starved bacteria. Given GSH’s multifaceted involvement in bacterial cellular processes and stress responses (18), pinpointing its exact contribution to recovery is challenging. Proteins, such as MetE (methionine biosynthesis) and DnaK (protein quality control), are known to be post-translationally modified via glutathionylation (i.e., the binding of GSH to cysteine residues), which protects them from permanent damage (26). Thus, irreversible damage to key proteins could potentially contribute to the compromised survival and recovery of N-starved Δ*gshAB* bacteria. GSH can be catabolized by γ-glutamyltransferase (GGT) into cysteine and glycine, thereby replenishing the intracellular amino acid pool (27). It is therefore plausible that, under N starvation, changes in amino acid availability may be responsible for the demise and impaired recovery of prolonged N-starved GSH-deficient bacteria. However, since bacteria lacking GGT (Δ*ggt*) recovered like WT bacteria (Fig. S2), this mechanism is unlikely to account for the compromised recovery phenotype of GSH-deficient bacteria. GSH is also important for aconitase function, the enzyme that converts citrate to isocitrate, the first step of the Krebs cycle. Thus, the absence of GSH induces oxidative damage to aconitase (28–30), which could cause the buildup of citrate and differentially impair metabolite flux through the Krebs cycle during growth recovery (from N and C starvation). Indeed, citrate levels in N-24 Δ*gshAB* bacteria are ~2.5-fold higher than in WT bacteria (Fig. S3). Furthermore, our results provide evidence of reduced cytoplasmic diffusibility in Δ*gshAB* bacteria, which could compromise the spatiotemporal rearrangement of macromolecules to enable efficient recovery.

In sum, our new results have uncovered additional roles for GSH in the adaptive response to N starvation that potentially extend its canonical function as a stress protectant. One such function involves the regulation of Hfq condensation dynamics during N starvation. More research, focused on the temporal glutathionylation dynamics of proteins at a systems-wide scale and targeted measurement of metabolites of the Krebs cycle in WT and Δ*gshAB* bacteria during N and C starvation, is needed to uncover the precise mechanistic basis by which GSH contributes to the adaptive response to N starvation.

## MATERIALS AND METHODS

### Bacterial strains and plasmids

All strains used in this study were derived from *Escherichia coli* K-12 and are listed in Table S1. Gene deletions were introduced into the WT and Hfq-PAmCherry strains as described previously (11). Briefly, the knockout alleles were transduced using the P1*vir* bacteriophage with strains from the Keio collection (31) serving as donors. Where multiple modifications were introduced into a strain, the existing *kanR* cassette was first cured by expressing the yeast *flp* flippase recombinase from pCP20 (32).

### Bacterial growth conditions

N starvation experiments were carried out as previously described in reference (19). Briefly, unless otherwise stated, bacterial cultures were grown in Gutnick minimal medium (33.8 mM KH_2_PO_4_, 77.5 mM K_2_HPO_4_, 5.74 mM K_2_SO_4_, and 0.41 mM MgSO_4_) supplemented with Ho-LE trace elements (33), 0.4% (wt/vol) glucose and 10 mM NH_4_Cl (for overnight cultures and recovery experiments) or 3 mM NH_4_Cl (for day cultures) at 37°C in a shaking (180 rpm) incubator. Bacterial day cultures for C starvation experiments were grown in Gutnick minimal media supplemented with 10 mM NH_4_Cl and 0.06% (wt/vol) glucose. For experiments containing HEX, HEX was added at 10% wt/vol, incubated for 30 min, and cells imaged on agarose pads containing 5% (wt/vol) HEX. For GSH addition experiments, GSH was added to a final concentration of 1 mM at *N*+. The proportion of viable cells in the bacterial population was determined by enumerating CFU/mL from serial dilutions on lysogeny broth agar plates.

### T7 phage infection assay

Bacterial cultures were grown in Gutnick minimal medium as described above to the indicated time points. Bacterial culture samples were taken, centrifuged, and resuspended in fresh Gutnick minimal media supplemented with either 2 mM NH_4_Cl and 12.5 mM glucose (for *N*+) or 5 mM glucose (for N-24) and diluted to *A*_600 nm_ of 0.3 to a final volume of 500 μL and transferred to a flat-bottomed 48-well plate, together with T7 phage at a final concentration of 4 × 10^9^ phage/mL. The cultures were then grown at 37°C with shaking at 700 rpm in a SPECTROstar Nano microplate reader (BMG LABTECH), and *A*_600 nm_ readings were taken every 10 min.

### Targeted metabolite measurement

At the indicated time point, approximately 10^10^ cells were collected and washed twice in ¼ strength Ringer’s solution (Thermo Scientific, BR0052G). Cell pellets were resuspended in 500 μL of cold (−20°C) methanol:acetonitrile:water (2:2:1, vol/vol/vol) + 0.1% formic acid. Samples were stored at −80°C until analysis. Before analysis, all vials received were reconstituted with 150 µL of 97.5% H_2_O + 2.5% acetonitrile + 0.2% formic acid (FA), diluted, vortexed, and transferred to inserts. Pooled quality control of all samples was then generated by pooling 10 µL of the first replicate for each experimental condition and injecting every 8 samples. All reagents used were of ultra-high-performance liquid chromatography (UHPLC) gradient grade, and all standards were of analytical grade. Targeted metabolomics analysis was performed using an Agilent 1290 Liquid chromatography (LC) system (Agilent Technologies, CA, USA) coupled to a QTRAP 4000 mass spectrometry (MS) system (SCIEX, Danaher, WA, USA). Chromatographic separation was achieved using a Luna Omega Polar C18 column (Phenomenex/Danaher, WA, USA). The analysis was conducted in positive ion mode (A: H2O + 0.2% FA/B: acetonitrile + 0.2% FA) on a 20-min gradient and in negative ion mode (A: H2O + 0.1% FA + 10 mM ammonium formate/B: 100% acetonitrile) on a 14-min gradient at 0.450 mL/min flow rate. All data were acquired in multiple reaction monitoring (MRM) mode.

Resulting spectra were analyzed using an in-house data analysis workflow based on reference (34).

### Immunoblotting

Immunoblotting was conducted in accordance with standard laboratory protocols, with primary antibodies incubated overnight at 4°C, and secondary antibodies incubated for 1 h at room temperature. The following antibodies were used: rabbit polyclonal anti-mCherry (Abcam ab167453) at 1:100 dilution, mouse monoclonal anti-RpoA (Biolegend, WP003) at 1:100 dilution, HRP goat anti-rabbit IgG (GE Healthcare NA934-1ML) at 1:10,000 dilution, and HRP goat anti-mouse IgG (Biolegend, 405,306) at 1:10,000 dilution. ECL Prime Western blotting detection reagent (GE Healthcare, RPN2232) was used to develop the blots, which were analysed on the ChemiDoc MP imaging system.

### Photoactivated localization microscopy and single-molecule tracking

For the PALM and single-molecular tracking (SMT) experiments, the Hfq-PAmCherry and mutant derivative reporter strains were used. Bacterial cultures were grown as described above, and samples were taken at the indicated time points, then imaged and analyzed as previously described (35, 36). Briefly, 1 mL of culture was centrifuged, washed, and resuspended in a small amount of Gutnick minimal medium supplemented with N and C concentrations that reflected the concentration contained in the media at the time point sampled. One microliter of the resuspended culture was then placed on a Gutnick minimal medium agarose pad (1% [wt/vol] agarose, 1× Gutnick minimal medium supplemented with N and C concentrations that reflected the concentration contained in the media at the time point sampled). Cells were imaged on a PALM-optimized Nanoimager (Oxford Nanoimaging, https://oni.bio/nanoimager/) with 15-millisecond exposures, at 66 frames per second over 10,000 frames. Photoactivatable molecules were activated using 405 nm and 561 nm lasers. Fields of view typically consisted of 100–200 bacterial cells.

For SMT, the Nanoimager software was used to localize the molecules by fitting detectable spots of high photon intensity to a Gaussian function. The Nanoimager software SMT function was then used to track individual molecules and draw trajectories of individual molecules over multiple frames, using a maximum step distance between frames of 0.6 μm and a nearest-neighbor exclusion radius of 0.9 μm. The software then calculated the apparent diffusion coefficients (D*) for each trajectory over at least four steps, based on the mean squared displacement of the molecule. To calculate %H_IM_, we collated D* values from multiple fields of view and determined the proportion of D* values that fell into our previously defined immobile population (D* ≤0.08 µm/s^2^) (16).

To calculate the proportion of cells with condensates, PALM data sets were first analyzed using the Cluster analysis function of CODI (Oxford Nanoimaging, https://alto.codi.bio/). Hfq condensates were analyzed using DBSCAN* with an eps distance of 75 nm and filtered to those with >50 localizations/cluster and a density >0.0015 localizations/nm^2^. Total number of condensates per field of view was then used to determine the proportion of the total cells that contained condensates.

To calculate the average D* of condensate and non-condensate associated Hfq molecules, the proportion of individual points within a single trajectory which localized within a condensate was calculated by using a custom Python script in combination with the DBSCAN* clustering data described above. To maximize stringency of this analysis, non-condensate associated trajectories were defined as those with 0 points localizing to a condensate, and condensate-associated trajectories were defined as those entirely localizing within a condensate.

## SUPPLEMENTAL MATERIAL

Supplemental figures and table jb.00012-26-s0001.docx Figures S1 to S3 and Table S1.

ASM does not own the copyrights to Supplemental Material that may be linked to, or accessed through, an article. The authors have granted ASM a non-exclusive, world-wide license to publish the Supplemental Material files. Please contact the corresponding author directly for reuse.
