# Correction for Ellis et al., “Glutathione impacts Hfq condensation in nitrogen-starved *Escherichia coli*”

**DOI:** 10.1128/jb.00294-26

**Published:** 2026-08-25

**Authors:** Harriet R. Ellis, Volker Behrends, Gerald Larrouy-Maumus, Josh McQuail, Sivaramesh Wigneshweraraj

## AUTHOR CORRECTION

Volume 208, no. 4, e00012-26, 2026, https://doi.org/10.1128/jb.00012-26. Introduction, paragraph 2, sentence 14: “We term these structures “condensates” because they form through a liquid-liquid phase separation-like process and are reversible, that is, they rapidly disperse upon N replenishment (see later)” should read “Work from the Amster-Choder laboratory identified Hfq-foci at the poles in cells experiencing osmotic stress and at late stationary phase (37, 38). The Hfq-foci are sensitive to hexanediol, suggesting that they form by liquid-liquid phase separation (39). Indeed, Goldberger et al. conclusively demonstrated *in vivo* and *in vitro* that the Hfq-foci form by liquid-liquid phase separation (38). Thus, hereon, we refer to the Hfq-foci as Hfq condensates.”

The following references were omitted.

37. Kannaiah S, Livny J, Amster-Choder O. 2019. Spatiotemporal organization of the *E. coli* transcriptome: translation independence and engagement in regulation. Mol Cell 76:574–589. https://doi.org/10.1016/j.molcel.2019.08.013

38. Goldberger O, Szoke T, Nussbaum-Shochat A, Amster-Choder O. 2022. Heterotypic phase separation of Hfq is linked to its roles as an RNA chaperone. Cell Rep 41:111881. https://doi.org/10.1016/j.celrep.2022.111881

39. McQuail J, Carpousis AJ, Wigneshweraraj S. 2022. The association between Hfq and RNase E in long-term nitrogen-starved *Escherichia coli*. Mol Microbiol 117:54–66. https://doi.org/10.1111/mmi.14782

